# Robotic versus open pancreatic surgery: a propensity score-matched cost-effectiveness analysis

**DOI:** 10.1007/s00423-022-02471-2

**Published:** 2022-03-21

**Authors:** Christian Benzing, Lea Timmermann, Thomas Winklmann, Lena Marie Haiden, Karl Herbert Hillebrandt, Axel Winter, Max Magnus Maurer, Matthäus Felsenstein, Felix Krenzien, Moritz Schmelzle, Johann Pratschke, Thomas Malinka

**Affiliations:** Department of Surgery, Campus Charité Mitte, Campus Virchow-Klinikum, Experimental Surgery and Regenerative Medicine, Charité – Universitätsmedizin Berlin, Augustenburger Platz 1, 13353 Berlin, Germany

**Keywords:** Robotic surgery, Pancreatic surgery, Cost analysis

## Abstract

**Background:**

Robotic pancreatic surgery (RPS) is associated with high intraoperative costs compared to open pancreatic surgery (OPS). However, it remains unclear whether several advantages of RPS such as reduced surgical trauma and a shorter postoperative recovery time could lead to a reduction in total costs outweighing the intraoperative costs. The study aimed to compare patients undergoing OPS and RPS with regards to cost-effectiveness in a propensity score-matched (PSM) analysis.

**Methods:**

Patients undergoing OPS and RPS between 2017 and 2019 were included in this monocentric, retrospective analysis. The controlling department provided financial data (costs and revenues, net loss/profit). A propensity score-matched analysis was performed or OPS and RPS (matching criteria: age, American society of anesthesiologists (ASA) score, gender, body mass index (BMI), and type of pancreatic resection) with a caliper 0.2.

**Results:**

In total, 272 eligible OPS cases were identified, of which 252 met all inclusion criteria and were thus included in the further analysis. The RPS group contained 92 patients. The matched cohorts contained 41 patients in each group. Length of hospital stay (LOS) was significantly shorter in the RPS group (12 vs. 19 days, *p* = 0.003). Major postoperative morbidity (Dindo/Clavien ≥ 3a) and 90-day mortality did not differ significantly between OPS and RPS (*p* > 0.05). Intraoperative costs were significantly higher in the RPS group than in the OPS group (7334€ vs. 5115€, *p* < 0.001). This was, however, balanced by other financial categories. The overall cost-effectiveness tended to be better when comparing RPS to OPS (net profit—RPS: 57€ vs. OPS: − 2894€, *p* = 0.328). Binary logistic regression analysis revealed major postoperative complications, longer hospital stay, and ASA scores < 3 were linked to the risk of net loss (i.e., costs > revenue).

**Conclusions:**

Surgical outcomes of RPS were similar to those of OPS. Higher intraoperative costs of RPS are outweighed by advantages in other categories of cost-effectiveness such as decreased lengths of hospital stay.

## Introduction

Laparoscopic surgery has been the established gold standard in the field of abdominal surgery for most procedures for several decades [1]. This also applies to hepatobiliary and pancreatic surgery, which was long considered the domain of open surgery [2–6]. One of the main reasons for the success of minimally invasive hepatobiliary surgery is the reduction of surgical trauma, leading to a shorter hospital stay and lower rates of postoperative complications [7, 8]. While laparoscopic surgery has become widely established for the treatment of liver tumors [9, 10], pancreaticoduodenectomies are still performed by most centers using conventional open surgery [11].

This dogma is currently undergoing a change, as robotic pancreatic surgery (RPS) is becoming increasingly established and significantly increases the feasibility and precision of distal pancreatectomies as well as pancreaticoduodenectomies [12, 13]. Recent reports show safe feasibility with comparable oncological outcomes (R0 rate) and low morbidity and mortality rates at high-volume centers [14, 15].

The potential benefits of minimally invasive robotic-assisted pancreatic surgery with faster patient recovery and potentially lower rates of postoperative complications such as wound dehiscence, pneumonia, and surgical site pain [16] are offset by the high costs of the procedure [17].

For many centers, these costs are the reason why these surgeries are not yet performed on a widespread basis. Nevertheless, it is important to determine whether a shortened postoperative recovery period and the associated cost savings will offset the costs incurred by the use of the surgical robot. Our group was able to show this, for example, for laparoscopic hemihepatectomies compared to open hemihepatectomies [18]. Reports comparing these outcomes of open, laparoscopic, and robotic pancreatic resections exist. However, most of them only analyze the cost-effectiveness of open, laparoscopic, and robotic distal pancreatectomies [6, 16, 19–21], whereas there is little evidence on the cost-effectiveness of robotic pancreaticoduodenectomies [22]. Furthermore, there is currently no evidence on the cost-effectiveness of RPS in Germany, where accounting is performed by applying the diagnose-related groups (DRG) system.

Since scheduling patients for either open or robotic surgery include a relevant selection bias, a one-to-one comparison of both approaches with regard to cost-effectiveness is not possible. Therefore, the present study aims to compare open and robotic-assisted pancreatic surgery with respect to direct and indirect costs using a propensity score-matched analysis and to evaluate the cost-effectiveness of robotic pancreatic surgery.

## Methods

### Patients and study design

The present study is a retrospective single-center analysis. All patients who underwent open or robotic partial pancreaticoduodenectomy (pylorus-preserving, PPPD, or Whipple’s procedure), distal pancreatectomy (DP), or total pancreatectomy (TP) at the Charité – Universitätsmedizin Berlin, Campus Charité-Mitte, and Campus Virchow-Klinikum in Berlin, Germany between 2017 and 2019 were included in the analysis. Of note, data from patients who underwent RPS were obtained and analyzed from a prospective database from the post-marketing CARE-Study (surgical assistance by robotic support; originally Chirurgische Assistenz durch Robotereinsatz, ethical approval code E/A4/084/17 (DRKS00017229)), which had been approved by the local ethics committee. The trial was funded by Intuitive Surgical, Inc. (Sunnyvale, California, United States).

**Fig. 1:**
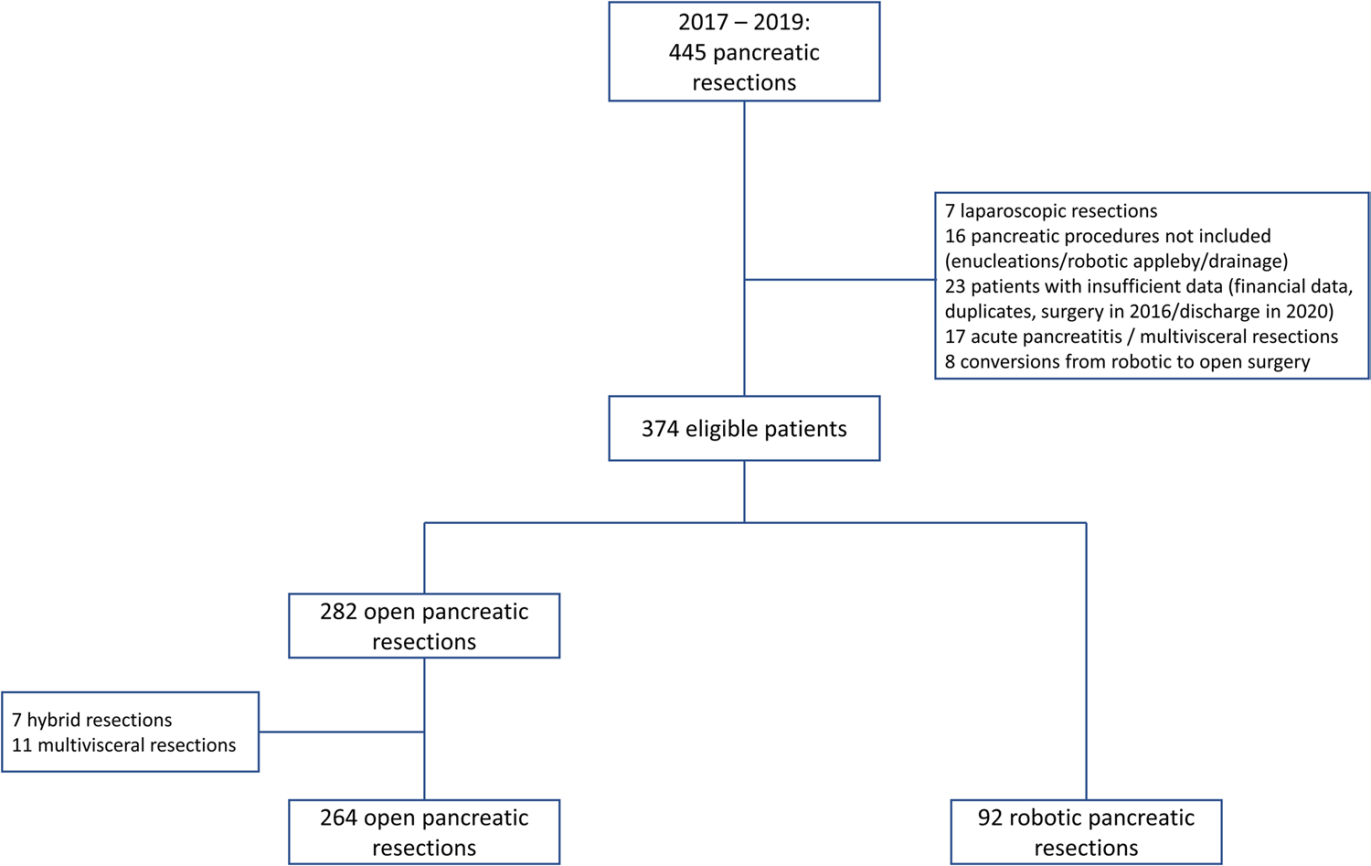
Flowchart for exclusion criteria

For further analysis, patients were divided into groups (1) OPS and (2) RPS. The inclusion criteria were RPS or OPS between January 2017 and December 2019; full financial data and medical history available. The exclusion criteria were patients who underwent procedures other than PPPD/Whipple’s procedure/DP/TP such as draining procedures (e.g., Partington-Rochelle), or enucleations; conversion from RPS to OPS; laparoscopic pancreatic and hybrid (laparoscopic + open) surgery; multivisceral resection (i.e., resections of three or more organs), concomitant colorectal resections; and major hepatectomy, respectively, patients who were operated in 2019 and were still hospitalized in 2020. Of note, an oral presentation which included parts of the data from the current report with different inclusion criteria was held in 2021 at the *Viszeralmedizin* congress in Leipzig, Germany [23]. Figure 1 shows the patient selection process.

### Cost analysis

Financial data were collected and provided by the controlling department of our center. The cost analysis consisted of two major branches: intra- and postoperative costs. Intraoperative costs contained expenses for operating theatre time per minute, presence of medical staff per minute (i.e., surgeons and anesthesiologists), surgical devices including staplers and cartridges, clips, tissue dissectors (e.g., vessel sealer, harmonic ace), sutures, and trocars. Postoperative costs included general costs for the intensive care units (ICU) and surgical wards. Moreover, we recorded costs for perioperative computed tomography (CT), magnetic resonance imaging (MRI), endoscopy, interventional therapies (e.g., CT-guided drainage), and perioperative transfusions. This led to the following categorization regarding costs and proceeds: (1) surgical ward, (2) intensive care unit, (3) dialysis, (4) surgery costs, (5) anesthesiology, (6) cardiology, (7) endoscopy, (8) radiology, (9) laboratory testing, (10) other diagnostic features, (11) therapeutic methods (e.g., physiotherapy, ergotherapy), (12) patient admission.

### Pre- and postoperative evaluation

Hospital admission took place 1 day before surgery, applying the concept of enhanced recovery after surgery (ERAS) according to the latest ERAS guidelines [24] with some modifications: Nasogastral tubes were placed in all cases after PD where a pancreaticojejunostomy was performed. The tubes were left until the 5th postoperative day and were eventually removed when the gastrointestinal passage X-ray showed no pathologies. The main ERAS elements including guidelines on preoperative biliary decompression, preoperative fasting, peridural anesthesia, postoperative nausea and vomiting (PONV) prophylaxis, early postoperative mobilization, and early postoperative nutrition were implemented as recommended. Perianastomotic/peripancreatic drains were placed routinely and were usually removed between the 3rd and 5th postoperative day in case lipase/bilirubin levels were not elevated. Patients who were scheduled for surgery all underwent routine preoperative workup including physical examination, laboratory testing (including carcinoembryonic antigen (CA) 19-9 and carbohydrate antigen (CEA) if indicated). Preoperative imaging included either CT or MRI scans. Preoperative imaging, as well as intraoperative findings, determined the type of pancreatic resection.

Postoperative complications were noted according to the Dindo/Clavien classification [25]. The International Study Group of Pancreatic Surgery (ISGPS) definitions for postoperative pancreatic fistula (POPF) and post-pancreatectomy hemorrhage (PPH) and delayed gastric emptying (DGE) were applied [26–28].

### Surgical technique

Pancreatic head resections (PPPD or Whipple’s procedures) were preferably performed as PPPD with standard lymphadenectomy. Standard reconstruction was either performed as pancreaticogastrostomy and pancreaticojejunostomy; biliary reconstruction was performed with a handsewn, retrocolic end-to-side hepaticojejunostomy.

DP was indicated in patients with lesions located in the body or tail of the pancreas. In cases of underlying/suspected malignancy, standard lymphadenectomy and splenectomy were performed as well. Patients with benign lesions underwent spleen-preserving DP, according to Kimura et al. [29].

Closure of the pancreatic remnant was either performed using the fish mouth-technique [30] or stapler closure (60-mm black cartridge, EndoGIA™, Medtronic, Minneapolis, MN, USA; reinforced by SEAMGUARD®, W.L. Gore, Flagstaff, AZ, USA).

OPS was performed by specialized hepatobiliary and pancreatic (HBP) surgeons. RPS was performed by the same team of two experienced pancreatic surgeons using the DaVinci® Xi surgical system (Intuitive Surgical Inc., Sunnyvale, CA, USA). Patients undergoing RPS were carefully selected. Exclusion criteria for RPS were severe chronic obstructive lung diseases with contraindication of pneumoperitoneum, suspected excessive intraabdominal adhesions (e.g., after multiple laparotomies, peritonitis), and suspected infiltration of big vessels requiring vascular resections. The oncological principles (lymphadenectomy/splenectomy in patients with underlying malignancies) were the same as in open surgery. The pancreas was dissected by electrocautery (PPPD/Whipple) or a stapling device (60-mm black cartridge, EndoGIA™, Medtronic, Minneapolis, MN, USA; reinforced by a bioabsorbable mesh: SEAMGUARD®, W.L. Gore, Flagstaff, AZ, USA). Reconstruction (pancreaticogastrostomy) was either performed via a small midline incision in the upper abdomen or completely minimally invasive.

The operative setup, port placement, and description of the surgical technique have recently been published by our group [31].

### Statistics

IBM SPSS Statistics for Macintosh Version 26.0 (IBM Corp., Armonk, NY, USA) was used for all calculations.

Continuous variables are displayed as median and range and statistically compared using the non-parametric Mann-Whitney *U* test. Counts/proportions are reported for categorical variables and statistically compared using the Pearson *χ*^2^ test was used. A binary logistic regression analysis was performed to identify independent risk factors for cost-ineffectiveness; findings are shown as odds ratio (OR) and 95% confidence interval (95% CI).

### Propensity score matching

We performed propensity score matching (PSM) analysis in order to balance possible confounders between OPS and RPS. We used R Studio Version 1.2.5033 (R Studio, Boston, MA, USA) to generate linear propensity score values (PSV) using the logistic regression method. The PSV were used to create matches with the nearest-neighbor matching method and a 1:1 ratio including replacement and a caliper of 0.2 of the standard deviation of the logit of the propensity score. The match is started from cases with the greatest propensity score. For propensity score matching (PSM), the following covariates were included in model age, American society of anesthesiologists (ASA) score (ASA 1–4), gender (male/female), body mass index (BMI), and type of pancreatic resection (PPPD or Whipple/distal pancreatectomy/total pancreatectomy). These baseline variables were selected as covariates due to (a) significant differences between the unmatched OPS/RPS groups and (b) because these variables potentially have a significant impact on important clinical outcome parameters such as morbidity, mortality, and duration of surgery. The surgical approach (RPS vs. OPS) was used as a dependent variable in the regression model.

## Results

### Patients’ characteristics

In total, 376 eligible patients could be identified during the study period, of which 282 underwent OPS and 92 underwent RPS (Figure 1). Compared to the RPS group, patients in the OPS group tended to be older (*p* = 0.004) and had more severe comorbidities (ASA 3 93% vs. 32%, *p* < 0.001, Table 1). Regarding the type of pancreatic surgery, there was a significant imbalance between the groups (*p* < 0.001, Table 1), DP was significantly more often performed in the RPS group (45% vs. 19%), whereas there were more TP in the OPS group (21% vs. 3%). After propensity score matching for age, BMI, gender, ASA score, and type of pancreatic resection, no significant differences were found in the respective variables. Both groups contained 41 patients after matching. Table 1 provides an overview of all patients’ characteristics including concomitant procedures before and after propensity score matching.

**Table 1:** Preoperative patient characteristics in open and robotic surgery groups before and after propensity score matching

	Open surgery *n* = 264	Robotic surgery *n* = 92	*P* value	Matched-open surgery *n* = 41	Matched-robotic surgery *n* = 41	*P* value
Age^1^	65 (22–88)	60 (22–87)	**0.004**	61 (22–85)	65 (37–87)	0.122
BMI^1^	24.6 (15.2–58.5)	25.1 (18.0–41.9)	0.598	25.1 (16.2–39.5)	25.6 (19.7–41.2)	0.806
Gender (male)^2^	150 (57)	49 (53)	0.554			
ASA score^2^			**< 0.001**			
1	0 (0)	3 (3)		0 (0)	0 (0)	1.000
2	15 (6)	60 (65)		12 (29)	12 (29)	
3	246 (93)	29 (32)		29 (71)	29 (71)	
4	3 (1)	0 (0)		0 (0)	0 (0)	
Type of resection^2^			**< 0.001**			0.407
PPPD/Whipple	168 (64)	48 (52)		29 (71)	27 (66)	
Distal pancreatectomy	40 (15)	41 (45)		9 (22)	13 (32)	
Total pancreatectomy	56 (21)	3 (3)		3 (7)	1 (2)	
Concomitant Procedures						
Splenectomy	59 (22)	38 (41)	**< 0.001**	1 (2)	0 (0)	0.314
(Partial) adrenalectomy	4 (2)	4 (4)	0.114	1 (2)	0 (0)	0.314
Gastric resections	5 (2)	1 (1)	0.605	1 (2)	1 (2)	1.000
Minor hepatic resection	5 (2)	1 (1)	0.605	9 (22)	11 (27)	0.607
Kidney resections	1 (0)	0 (0)	0.554	2 (5)	0 (0)	0.152
Vascular reconstruction	18 (7)	0 (0)	**0.010**			
Indication^2^			0.071			0.100
Periampullary carcinoma	37 (14)	19 (21)		2 (5)	10 (24)	
Pancreatic ductal adenocarcinoma	158 (60)	41 (45)		23 (56)	21 (51)	
Benign lesions	54 (21)	28 (30)		10 (24)	8 (20)	
Neuroendocrine tumors	4 (2)	2 (2)		2 (5)	1 (2)	
Others	11 (4)	2 (2)		4 (10)	1 (2)	

^1^Data are presented as median and range, ^2^data is presented as count and proportions (%); *PPPD*, pylorus-preserving pancreaticoduodenectomy

### Perioperative details

RPS procedures were shorter than OPS procedures (262 vs. 313 minutes, *p* < 0.001), these differences were not present anymore after matching (*p* = 0.164, Table 2). ICU stay was comparable in both groups before and after matching. However, the total hospital stay in days was shorter in the RPS group both before and after matching (*p* < 0.001 and 0.003, respectively, Table 2). Major complications (Dindo/Clavien > grade II) were more frequent in the RPS group before matching (55% vs. 41%, *p* = 0.014). Pancreas-specific morbidity was significantly higher (POPF and PPH, respectively, both *p* < 0.05). These differences could not be observed after propensity score matching except for delayed gastric emptying (*p* = 0.048). Table 2 shows the perioperative details both before and after matching.

**Table 2:** Perioperative details in open and robotic surgery groups before and after propensity score matching

	Open surgery *n* = 264	Robotic surgery *n* = 92	*P* value	Matched-open surgery *n* = 41	Matched-robotic surgery *n* = 41	*P* value
Duration of surgery^1^	315 (96–576)	261.5 (62–535)		315 (123–447)	271 (111–532)	0.164
ICU stay (days)^1^	2 (0–84)	1 (1–43)		1 (0–13)	1 (1–43)	0.401
Hospital stay (days)^1^				19 (8–55)	12 (6–91)	**0.003**
Readmission to ICU^2^	47 (18)	18 (20)	0.706	7 (17)	8 (20)	0.775
Postoperative pancreatic fistula (POPF)^2^			**0.001**			0.108
Grade A	6 (2)	3 (3)		5 (12)	1 (6)	
Grade B	31 (12)	27 (29)		5 (12)	11 (27)	
Grade C	1 (0)	1 (1)		0 (0)	1 (2)	
Delayed gastric emptying			0.327			**0.048**
Yes	20 (8)	10 (11)		1 (2)	6 (15)	
No	244 (92)	82 (89)		40 (98)	35 (85)	
Post-pancreatectomy hemorrhage (PPH)^2^			**< 0.001**			0.258
Grade A	20 (8)	3 (3)		0 (0)	1 (8)	
Grade B	7 (3)	27 (2)		5 (83)	11 (85)	
Grade C	2 (1)	1 (1)		1 (17)	1 (8)	
Complications (Dindo/Clavien)^2^			**< 0.001**			0.051
None	97 (37)	29 (32)		11 (27)	15 (37)	
I	28 (11)	6 (7)		7 (17)	3 (7)	
II	31 (12)	6 (7)		6 (15)	2 (5)	
IIIa	50 (19)	8 (9)		9 (22)	3 (7)	
IIIb	23 (9)	25 (27)		3 (7)	10 (24)	
IVa	7 (3)	13 (14)		1 (2)	5 (12)	
IVb	13 (5)	2 (2)		2 (5)	2 (5)	
V	15 (6)	3 (3)		2 (5)	1 (2)	
Major complications (> grade II)^2^	108 (41)	51 (55)	**0.016**	17 (42)	21 (51)	0.376
90-day mortality^2^	4 (2)	3 (3)	0.299	0 (0)	1 (1)	0.314

^1^Data are presented as median and range, ^2^data is presented as count and proportions (%)

### Costs and proceeds after OPS and RPS

Regarding the costs for OPS and RPS, there were significant differences in numerous categories. ICU costs were significantly lower in the RPS group (907€ vs. 2629€, *p* < 0.001, Table 3), whereas surgery costs (such as operating room time, staff costs, materials) were significantly higher when RPS was performed (7092€ vs. 4881€, *p* < 0.001). Costs for anesthesiology, laboratory tests, therapeutic methods as well as patient admission were lower in the RPS costs (all *p* < 0.05, Table 3). Total costs were comparable in both groups (OPS: 21,933€, RPS 20,907€, *p* = 0.305). With regard to proceeds, there were significant differences in the categories ICU, surgery proceeds, endoscopy, radiology, laboratory tests, other diagnostic features, therapeutic methods, and patient admission (all *p* < 0.001, Table 3). This led to a significantly higher net profit in the OPS group (+ 151€ vs. − 912€, *p* = 0.039, Table 3).

**Table 3:** Costs and proceeds in open and robotic surgery before propensity score matching

	Open surgery *n* = 264	Robotic surgery *n* = 92	*P* value
Costs in €			
Surgical ward	7227 (504–50,603)	6078 (1736–34,287)	0.116
Intensive care unit	2604 (438–161,309)	907 (0–69,460)	**< 0.001**
Dialysis	0 (0–27,334)	0 (0-8,416)	0.486
Surgery costs	4861 (2242–18,867)	7093 (2971–17,724)	**< 0.001**
Anesthesiology	2466 (1054–12,340)	2183 (711–8,806)	**0.001**
Cardiology	0 (0–10,176)	0 (0–0)	0.184
Endoscopy	0 (0–32,785)	0 (0–9600)	0.872
Radiology	587 (0–22,893)	477 (0–11,486)	0.399
Laboratory tests	1723 (297–32,729)	1224 (346–12,519)	**< 0.001**
Other diagnostic features	31 (0–1171)	21 (0–840)	0.094
Therapeutic methods (e.g., physiotherapy, ergotherapy)	254 (0–3532)	209 (0–2891)	**0.040**
Patient admission	0 (0–85)	0 (0–0)	0.403
Total costs in €	21,933 (7665–256,937)	20,907 (8612–135,373)	0.344
Proceeds in €			
Surgical ward	5700 (1583–14,824)	5607 (1191–9129)	0.190
Intensive care unit	4474 (865–176,602)	4399 (1016–91,007)	**0.002**
Dialysis	0 (0–21,538)	0 (0–8947)	0.481
Surgery proceeds	6068 (1389–21,888)	4558 (1389–14,603)	**< 0.001**
Anesthesiology	2261 (484–8206)	1699 (614–4365)	**< 0.001**
Cardiology	10 (0–12,419)	8 (1–2141)	0.479
Endoscopy	321 (0–2103)	413 (161–1598)	0.823
Radiology	444 (233–17,025)	531 (261–4606)	0.098
Laboratory tests	1362 (344–25,122)	1264 (548–9260)	**0.001**
Other diagnostic features	215 (19–1361)	172 (80–1136)	**< 0.001**
Therapeutic methods (e.g., physiotherapy, ergotherapy)	286 (0–2850)	322 (0–1573)	**0.004**
Patient admission	97 (0–612)	98 (0–482)	**< 0.001**
Total proceeds in €	21,705 (9510–238,681)	21,479 (9510–127,283)	**0.001**
**Net profit/loss in € (min/max)**	**+ 213 (− 34,019/+ 35,891)**	**− 912 (− 34,289/+ 11,815)**	**0.028**

All data are presented as median and range, currency: Euro

**Fig. 2:**
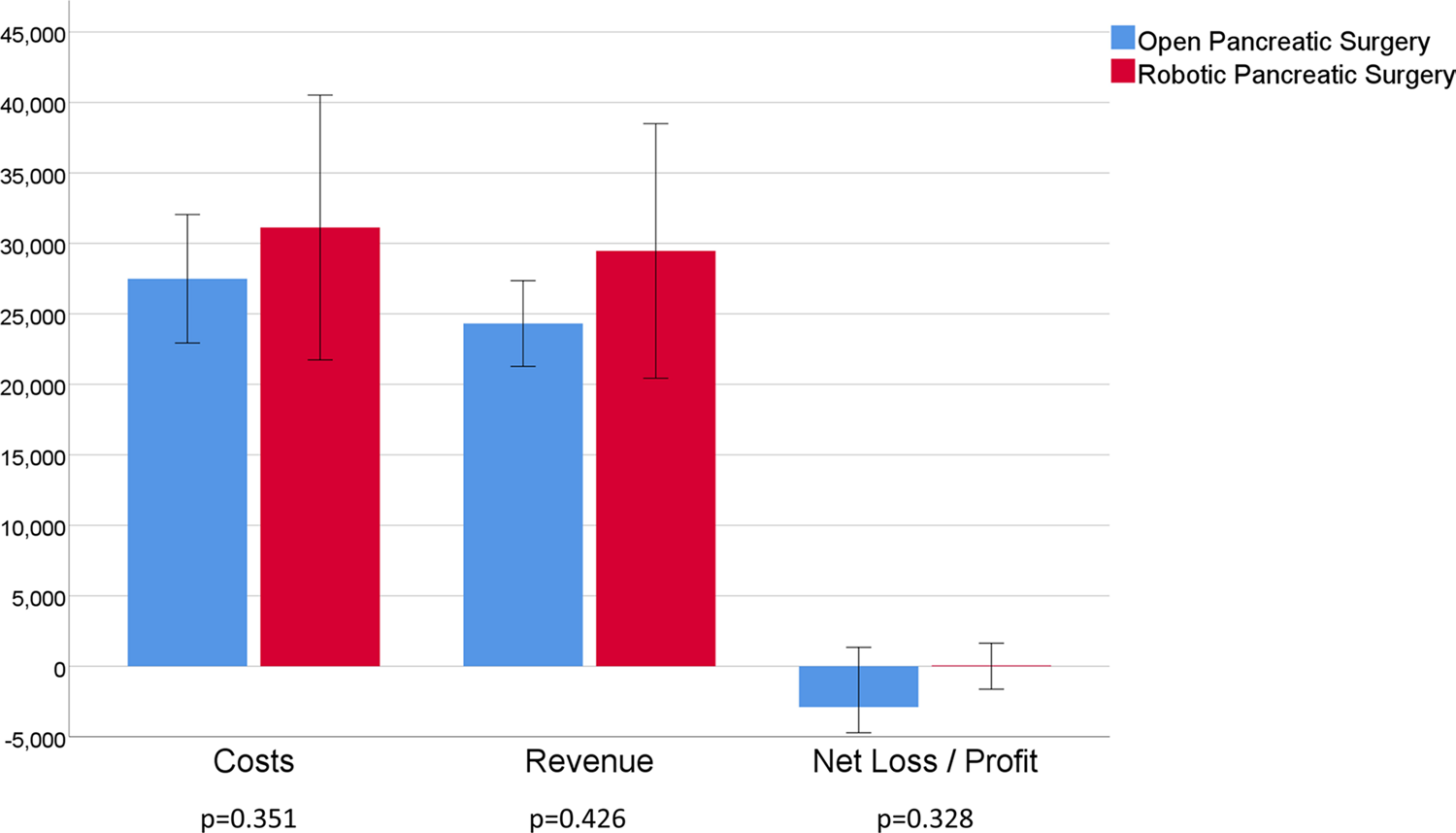
Median costs, revenues, and net profit/loss after open and robotic pancreatic surgery. Error bars: 95% confidence interval

After propensity score matching, costs were found to be higher for OPS in the categories surgical ward, radiology, and laboratory tests (all *p* < 0.05, Table 4). Surgery-associated costs were higher in the RPS group (7334€ vs. 5115€, *p* < 0.001, Table 4). Proceeds for cardiology, other diagnostic features, therapeutic methods, and patient admission were all below 1000€ but significantly higher in the RPS group (all *p* < 0.05, Table 4). After matching, median net profit tended to be higher in the RPS group; however, the differences were short of statistical significance (Table 4, Figure 2). Figure 2 shows the total costs, total proceeds as well as net profit/loss for both groups.

**Table 4:** Costs and proceeds in open and robotic surgery after propensity score matching

	Matched-open surgery *n* = 41	Matched-robotic surgery *n* = 41	*P* value
Costs in €			
Surgical ward	8293 (1579–26,744)	6161 (1736–34,287)	**0.032**
Intensive care unit	2088 (456–20,549)	945 (319–69,460)	0.163
Dialysis	0 (0–3363)	0 (0–6223)	0.926
Surgery costs	5115 (2271–16,925)	7334 (4979–17,724)	**< 0.001**
Anesthesiology	2450 (1317–10,344)	2367 (1044–6354)	0.626
Cardiology	0 (0–0)	0 (0–0)	1.000
Endoscopy	0 (0–8354)	0 (0–9600)	0.223
Radiology	862 (49–5899)	425 (48–11,048)	**0.029**
Laboratory tests	1722 (468–8110)	1192 (486–11,032)	**0.007**
Other diagnostic features	30 (0–374)	40 (0–840)	0.948
Therapeutic methods (e.g., physiotherapy, ergotherapy)	234 (0–1460)	188 (0–2891)	0.531
Patient admission	0 (0–522)	0 (0–179)	0.086
Total costs in €	22,241 (11,922–77,056)	20,138 (12,007–120,055)	0.351
Proceeds in €			
Surgical ward	5722 (3277–11,852)	5607 (3072–9129)	0.539
Intensive care unit	4474 (1016–26,403)	4583 (1740–91,007)	0.504
Dialysis	0 (0–2807)	0 (0–3164)	0.926
Surgery proceeds	6068 (1389–1198)	6144 (1676–14,603)	0.253
Anesthesiology	2294 (614–4573)	2,122 (715–4365)	0.132
Cardiology	25 (3–516)	6 (1–2141)	**0.002**
Endoscopy	321 (150–1038)	416 (161–1598)	0.163
Radiology	531 (261–2880)	531 (261–4606)	0.657
Laboratory tests	1362 (548–7279)	1362 (623–8525)	0.481
Other diagnostic features	220 (80–1143)	172 (80–727)	**0.001**
Therapeutic methods (e.g., physiotherapy, ergotherapy)	277 (0–879)	344 (0–1572)	**0.028**
Patient admission	94 (0–183)	98 (0–482)	**0.003**
Total proceeds in €	21,958 (9510–5830)	22,147 (11,239–127,283)	0.426
**Net profit/loss in € (min/max)**	**− 2894 (− 33,911/+ 10,985)**	**57 (− 34,289/+ 11,816)**	0.328

All data are presented as median and range, currency: Euro

### Risk factors for net loss after pancreatic surgery

To identify independent risk factors for net loss after pancreatic surgery, a binary logistic regression analysis was performed (Table 5). Of 374 patients, costs exceeded revenues in 194 patients (52%), resulting in net loss. Regression analysis revealed that major complications (Dindo/Clavien > grade II, *p* < 0.001), a longer hospital stay (*p* = 0.015), and ASA score < 3 (*p* = 0.040) were independent risk factors for net loss (Table 5).

**Table 5:** Multivariate logistic regression analysis of independent risk factors for net loss after pancreatic surgery

All cases with a net loss after pancreatic surgery (*n* = 184/356)		
Variable	Odds ratio (95% confidence interval)	*P* value
Robotic surgery	0.948 (0.449–2.000)	0.889
Female gender	0.714 (0.445–1.145)	0.162
Age (years)	0.995 (0.976–1.014)	0.995
BMI (kg/m^2^)	0.993 (0.949–1.040)	0.993
Major complications (> grade II)	3.172 (1.857–5.420)	**< 0.001**
Intensive care unit stay (days)	1.009 (0.966–1.053)	0.695
Hospital stay (days)	0.967 (0.941–0.994)	**0.015**
ASA score 3 or 4	0.434 (0.195–0.965)	**0.041**
Distal pancreatectomy	1.314 (0.718–2.404)	0.376

*BMI*, body mass index; *ASA*, American Society of Anesthesiologists

## Discussion

Patients who are scheduled for robotic pancreatic surgery are highly selected according to various patient characteristics such as age, BMI, and comorbidities, which makes the comparison of the cost-effectiveness of this approach difficult. This was confirmed in the present study; patients undergoing RPS were significantly younger and had fewer comorbidities than patients scheduled for OPS. We, therefore, performed propensity score matching, after which there were no differences in patient characteristics. The comparison of costs and proceeds before matching showed clear advantages of OPS over RPS, which was not evident anymore after matching. The higher intraoperative costs of RPS were compensated in particular by a reduced length of hospital stay.

The perioperative data from our two cohorts presented here, including operative time, postoperative morbidity and mortality, are comparable to those from previous studies [22, 32–34]. Pancreas-specific morbidity (PPH, POPF, and DGE) were higher in the unmatched cohorts but tended to be similar in the matched cohorts. We thus conclude that the differences that were found in the unmatched cohorts are likely to be due to differences in the study populations that are not existent anymore after matching.

Today, there are various studies examining the cost-effectiveness of RPS [6, 16, 19–22, 32, 34–39]. Nonetheless, the generalized comparability of these studies is not easy since some of these studies are from different countries with different health systems and currencies. Furthermore, most of them focus on DP procedures only [6, 16, 19, 20, 34, 36–38], of which some merely compared robotic and laparoscopic DP [21, 34, 36, 37]. Most authors agree that robotic DP is of advantage with regard to the length of hospital stay as well as perioperative costs [19, 20], which is in line with the results of the present study. We furthermore found significantly lower costs for postoperative imaging (“radiology”) and laboratory tests.

Baker et al. compared the perioperative outcomes and costs of open and robotic PD. They found no significant differences in severe morbidity and postoperative mortality between the groups. Intraoperative costs were higher for RPS, but total costs did not differ significantly between RPS and OPS [22]. Kowalsky and colleagues found significantly better cost-effectiveness in patients who underwent robotic PD when the ERAS pathway was implemented. ERAS also led to a significantly shorter hospital stay in patients who underwent RPS. This effect was not present in patients who underwent OPS [39]. We were not able to examine this effect since ERAS was the standard approach for all patients. Nonetheless, it is likely that the positive effect of the ERAS program is also one of the reasons for the good results of the RPS group in the present study.

In our analysis, the largest and most significant difference between the costs for OPS and RPS were operative costs. This is in line with the findings from other studies [19, 22]. An aspect that is unique when compared to previous studies assessing the cost-effectiveness of RPS and OPS is the fact that we were able to identify factors that were independently associated with cost-ineffectiveness (i.e., net loss). Besides major complications and length of hospital stay, we found that lower ASA scores (1 and 2) were associated with a significantly higher risk for a net loss. This can be explained by the fact that comorbidities are known to trigger higher DRG classes and increase reimbursements by insurance companies [40].

The present study has some limitations, such as its retrospective nature leading to potential bias. Furthermore, group sizes are not equal before matching, which is due to the fact that RPS is not an eligible approach for all patients. Nonetheless, this is the first propensity score-matched cohort study comparing costs and profits after OPS and RPS, respectively, in Germany and other countries where reimbursement by health insurers is based on the DRG system. In addition to patient-specific differences such as age and ASA score, that can be overcome by matching, there is another potential selection bias. This bias is due to tumor-specific differences such as locally advanced tumors which are generally not eligible for RPS. Also, there were differences in operating surgeons between RPS and OPS that might potentially impact the outcome. Another important issue is the fact that there were—despite propensity score matching—there were some non-significant differences between the OPS and RPS groups that could not be overcome. The slightly higher proportion of pancreatic head resections as well as slightly more malignant tumors in the OPS groups may be a bias influencing morbidity and mortality rates [41, 42].

One of the main strengths of the present study compared to previous studies is that we did compare not only the total costs but also the revenues that were reimbursed by the health insurance companies. This allows us to truly compare the cost-effectiveness of both approaches in the German DRG system.

## Conclusions

The present study shows that RPS does not only lead to comparable surgical outcomes when compared to OPS but also significantly reduces the median hospital stay. This, in turn, substantially reduces the periprocedural costs of RPS. Despite the significantly higher intraoperative costs for RPS, median overall net profit tended to be higher in RPS when compared to OPS. In conclusion, the higher intraoperative costs of RPS are outweighed by advantages in other categories of cost-effectiveness and should be favored in selected patients and specialized centers.
